# Comorbid anxiety-like behavior in a rat model of colitis is mediated by an upregulation of corticolimbic fatty acid amide hydrolase

**DOI:** 10.1038/s41386-020-00939-7

**Published:** 2021-01-15

**Authors:** Haley A. Vecchiarelli, Maria Morena, Catherine M. Keenan, Vincent Chiang, Kaitlyn Tan, Min Qiao, Kira Leitl, Alessia Santori, Quentin J. Pittman, Keith A. Sharkey, Matthew N. Hill

**Affiliations:** 1 Neuroscience Graduate Program, University of Calgary, Calgary, AB T2N4N1 Canada; 2 Hotchkiss Brain Institute, University of Calgary, Calgary, AB T2N4N1 Canada; 3 Mathison Centre for Mental Health Research and Education, University of Calgary, Calgary, AB T2N4N1 Canada; 4 Department of Cell Biology and Anatomy, University of Calgary, Calgary, AB T2N4N1 Canada; 5 Department of Psychiatry, University of Calgary, Calgary, AB T2N4N1 Canada; 6 Snyder Institute for Chronic Diseases, University of Calgary, Calgary, AB T2N4N1 Canada; 7 Department of Physiology and Pharmacology, Cumming School of Medicine, University of Calgary, Calgary, AB T2N4N1 Canada

**Keywords:** Experimental organisms, Stress and resilience, Limbic system, Behavioural methods, Anxiety

## Abstract

Peripheral inflammatory conditions, including those localized to the gastrointestinal tract, are highly comorbid with psychiatric disorders such as anxiety and depression. These behavioral symptoms are poorly managed by conventional treatments for inflammatory diseases and contribute to quality of life impairments. Peripheral inflammation is associated with sustained elevations in circulating glucocorticoid hormones, which can modulate central processes, including those involved in the regulation of emotional behavior. The endocannabinoid (eCB) system is exquisitely sensitive to these hormonal changes and is a significant regulator of emotional behavior. The impact of peripheral inflammation on central eCB function, and whether this is related to the development of these behavioral comorbidities remains to be determined. To examine this, we employed the trinitrobenzene sulfonic acid-induced model of colonic inflammation (colitis) in adult, male, Sprague Dawley rats to produce sustained peripheral inflammation. Colitis produced increases in behavioral measures of anxiety and elevations in circulating corticosterone. These alterations were accompanied by elevated hydrolytic activity of the enzyme fatty acid amide hydrolase (FAAH), which hydrolyzes the eCB anandamide (AEA), throughout multiple corticolimbic brain regions. This elevation of FAAH activity was associated with broad reductions in the content of AEA, whose decline was driven by central corticotropin releasing factor type 1 receptor signaling. Colitis-induced anxiety was reversed following acute central inhibition of FAAH, suggesting that the reductions in AEA produced by colitis contributed to the generation of anxiety. These data provide a novel perspective for the pharmacological management of psychiatric comorbidities of chronic inflammatory conditions through modulation of eCB signaling.

## Introduction

In peripheral inflammatory conditions, such as inflammatory bowel diseases (IBD), comorbid anxiety and depression are associated with increased disease activity, greater rate of relapse and reduced responsiveness to therapies [1–5], significantly reducing patient quality of life [6, 7]. It is established in cohorts from around the world that patients with IBD (ulcerative colitis and Crohn’s disease; combined, and each, separately,) show a 2–3 times greater incidence of anxiety and depression [2–4, 8–29]. It is likely that the driving force behind these psychiatric comorbidities is disease activity [8, 13, 30–35], implying, at least partially, that inflammation and a dysregulation of the gut-brain axis may be involved in the pathogenesis of psychiatric comorbidities in IBD. Both basally and during disease states, the gut-brain axis allows for bidirectional communication between the brain and the gut, including at the levels of the autonomic nervous system and circumventricular organs [36]. As such, understanding the neural mechanisms that underlie the generation of anxiety and depression in peripheral inflammatory disorders may allow for the development of novel treatment approaches to manage these comorbid symptoms that severely impact individuals with these disorders.

Peripheral inflammation, particularly within the gut, is known to be a potent activator of the hypothalamic-pituitary-adrenal (HPA) axis [37, 38]. Sustained elevations in circulating glucocorticoid hormones can modulate central processes, including those involved in the regulation of emotional behavior [39, 40]. One system known to be sensitive to hormonal components of the HPA axis, and that is a significant regulator of emotional behavior, is the endocannabinoid (eCB) system [41–43].

Constitutive eCB signaling constrains anxiety, as acute pharmacological disruption of eCB function rapidly produces a state of anxiety [44–46]. Similarly, exposure to stress is known to increase activity of the enzyme fatty acid amide hydrolase (FAAH), which metabolizes the eCB ligand anandamide (AEA) [47–49], through the release of the neuropeptide corticotropin-releasing factor (CRF; alternatively corticotropin-releasing hormone (CRH)) and subsequent activation of the CRF type 1 receptor (CRF-R1) [50]. This suppression of AEA signaling by CRF-R1 activity promotes the development of anxiety, largely through coordinated actions in corticolimbic circuits encompassing the amygdala [50], medial prefrontal cortex [51], and hippocampus [52]. Interestingly, CRF signaling is also known to be important for the development of anxiety in response to inflammation, as blockade of CRF signaling can dampen anxiety and other adverse behavioral responses to a variety of experimental inflammatory conditions such as cerebral ischemia [53], arthritis [54, 55], and inflammatory pain [54, 56, 57]. As sustained inflammation is known to produce an upregulation of central CRF [57–59], it seems plausible that this could result in a suppression of AEA signaling that in turn could contribute to the development of comorbid anxiety in colitis.

To further examine the relationship between eCBs and peripheral inflammation, we utilized a rat model of colitis to investigate the potential role that the eCB system plays in the mechanisms underlying psychiatric comorbidity in chronic inflammatory diseases. Colitis represents an ideal condition for this investigation, as humans afflicted with colitis exhibit considerable psychiatric comorbidities, particularly anxiety [1–5], and antagonism of CRF-R1 in humans with IBD has been found to normalize both alterations in neural connectivity and changes in emotional behavior [60, 61]. Rodent models of colitis produce a sustained state of systemic inflammation [62–65], exhibit upregulation of central CRF [66–69] and recapitulate the anxiety phenotype [70–73] seen in the human condition, making them an ideal model to explore the role of eCBs in these processes.

## Methods and materials

### Animals

All experiments utilized adult (~300–350 g at time of colitis induction), male or female, Sprague Dawley rats from Charles River (Saint Constant, QC, Canada, RGD Cat# 734476; RRID:RGD_734476). Animals were allowed to acclimate for at least one week prior to experiment onset. Rats were paired-housed under specified pathogen free conditions on a 12:12 h light/dark cycle with *ad libitum* access to food and water. All experiments were conducted during the light phase of the cycle. All animal protocols were approved by the University of Calgary Animal Care Committee and followed guidelines from the Canadian Council for Animal Care. For each set of experiments described below, animals from a minimum of 2, and up to 4, cohorts were used, aside from locomotor activity which was assessed in a single cohort.

### Colitis induction and assessment

Under brief isoflurane anesthesia, rats received an intracolonic bolus of 2,4,6-trinitrobenzenesulfonic acid (TNBS) (Millipore Sigma, Darmstadt, Germany, #92822; 0.45 mL, 50 mg/mL, 50% [vol/vol] in ethanol/water), via a cannula, inserted 7 cm proximal to the anus [74–77]. TNBS haptenizes self and microbial proteins, which makes them available to initiate an immune response in the host’s own immune system [78–80]. Control animals received the same volume of saline delivered similarly, as is standard in the field. Body weight was monitored. Behavioral testing took place 1-week after the induction of colitis after which rats were euthanized by decapitation. Colons were quickly removed, rinsed with ice-cold physiological saline (0.9%) and cut open longitudinally to enable macroscopic scoring for damage and inflammation, including adhesions, diarrhea and degree of ulceration. This score was adapted from those previously reported [74, 77] and is described in the Supplementary Materials. An ~100 mg sample of colon was excised, snap frozen and stored at −80 °C until assayed for myeloperoxidase (MPO) activity, as previously described [74–77] and in the Supplementary Materials.

### Behavioral measures

#### Locomotor activity

Ambulatory activity was assessed using the Opto-Varimex-5 Auto Track (Columbus Instruments, Columbus, OH, USA) infrared beam activity monitor with a 17.5”x17.5” arena as previously described [81]. Day 0 testing occurred prior to TNBS or saline administration. Data were normalized within an animal to a percentage of its Day 0 activity.

#### Elevated plus maze (EPM)

Animals were subjected to handling and body weight measurement in the behavior testing room at least 5 days prior to anxiety testing. EPM (Med Associates, Fairfax, VT, USA) testing occurred on Day 7 following colitis induction under dim light and with a white noise background. EPM was performed for 5 min as previously described [82] and is detailed in the Supplementary Materials.

### Biochemical and molecular measures

#### Corticosterone enzyme-linked immunosorbent assay (ELISA)

After behavioral experiments were completed on Day 7 after the induction of colitis, trunk blood was collected as previously described [83, 84] and plasma corticosterone levels were assayed using a commercially available ELISA kit (Cayman Chemical Company, Ann Arbor, Michigan, USA, #500655), according to the manufacturer’s protocol.

#### Endocannabinoid measurements

Excisions of brain structures were performed on ice as described previously [85] and samples were immediately snap frozen and stored at −80 °C. Analysis of AEA, and the other primary eCB 2-arachidonoylglycerol (2-AG), was conducted through liquid chromatography/tandem mass spectrometry on an Eksigent ekspert micro liquid chromatographer 200 coupled to an AB Sciex Qtrap 5500 mass spectrometer (SCIEX, Framingham, MA, USA) as previously described [82, 86] and in the Supplementary Materials.

#### Enzyme activity assays

Brain structures were excised on ice [85] and samples were then immediately snap frozen and stored at −80 °C. Brain tissues were homogenized and membrane fractions were isolated as described previously [50].

The activity of the enzyme FAAH, which is responsible for the degradation of AEA, was measured as the conversion of [^3^H]-AEA to [^3^H]-ethanolamine [87]. Similarly, monoacylglycerol lipase (MAGL) activity was measured as the conversion of [^3^H]-2-oleoylglycerol (2-OG) to [^3^H]-glycerol [88]. The maximal hydrolytic activity (V_max_) of FAAH and MAGL and the binding affinities (K_m_) of AEA for FAAH and 2-AG for MAGL were determined by fitting the data to the Michaelis-Menten equation using Prism v8 (GraphPad, San Diego, CA, USA, RRID:SCR_002798).

#### Gene expression analysis

mRNA isolation and cDNA synthesis was performed as previously described [50, 83, 84] and detailed in the Supplemental Materials, using magnetic bead homogenization with a TissueLyser LT (Qiagen, Hilden, Germany) and the RNeasy Plus Universal Mini Kit (Qiagen, #73404) on a Qiacube (Qiagen, RRID:SCR_018618) followed by the QuantiTect Reverse Transcription Kit (Qiagen, #205314) according to the manufacturer’s protocols. Primers for genes of interest were designed using IDTDNA PrimerQuest (Coralville, Iowa, USA) and acquired from IDTDNA (Table S1). qPCR was performed as previously described (1 min at 90 °C, 40 cycles of 95 °C for 5 s and 60 °C for 30 s, before a final melt step) using PerfeCTa SYBR Green Fast Mix (QuantaBio, Beverly, MA, USA, #95072) on a RotoGene Q light cycler (Qiagen). Data were analyzed using the 2^−ΔΔCT^ method. Data were normalized so the average of the saline group was 1.

### Pharmacological intervention - behavioral studies

As global FAAH inhibition is associated with suppression of colonic inflammation [89–97], we administered the FAAH inhibitor intracerebroventricularly (icv) to be able to establish the importance of central FAAH inhibition on colitis-induced anxiety. Rats underwent intracranial cannulations as previously described [50]. Briefly, under isoflurane anesthesia and analgesic treatment (meloxicam (1 mg·kg^−1^, subcutaneously)), rats were implanted with a 12 mm unilateral cannula into the lateral ventricle (coordinates: −0.90 mm anteroposterior, 1.4 mm mediolateral, and −2.8 mm dorsoventral from Bregma). Rats were given one week of recovery before colitis induction, and as in the previous experiments, anxiety-like behavior was tested 7 days later. On the three consecutive days before drug infusion and testing, rats were exposed to daily mock infusions. Two hours prior to EPM testing, animals received icv infusions (2 µL; 1 µL/min) of solutions containing vehicle (0.9% saline:dimethylsulfoxide (DMSO):Tween-80 [80:10:10; vol:vol:vol]) or a FAAH inhibitor (PF-04457845 (PF); Pfizer, New York, NY, USA; 100 ng and 1 µg) [98–100]. Infusers extended 2 mm past guide cannula and were left in place 1 min following infusion. Two hours following drug administration all animals were tested for 5 min in the EPM as described above. Following testing, animals were euthanized and ventricular cannula placement was confirmed with dye infusion post-mortem.

### Pharmacological intervention - biochemical studies

To understand the role of CRF signaling on the colitis-induced reductions of AEA signaling, we examined the impact of sustained disruption of CRF-R1 signaling during the entire duration of colitis (i.e., 7 days) utilizing continuous drug infusion with an osmotic mini-pump (Alzet, Cupertino, CA, USA; Model 2002; 0.5 µL/h) connected to a 5 mm cannula (Alzet, Brain Infuser Kit 2) [101]. The osmotic mini-pumps were pre-loaded with vehicle (artificial cerebral spinal fluid [102]: DMSO [90:10; vol:vol]) or a CRF-R1 antagonist (antalarmin (Cayman Chemical Company, 15147); 10 µg/day) [103] and were incubated at 37 °C submerged in sterile physiological saline for 1–3 days prior to implantation. Under isoflurane anesthesia and analgesic (meloxicam (1 mg·kg^−1^, subcutaneously)) treatment, the unilateral cannula was placed into the lateral ventricle, −0.90 mm anteroposterior and 1.4 mm mediolateral from Bregma, and the pump was placed subcutaneously. Surgeries were performed on the same day, but prior to, TNBS or saline administration. One week following surgery and colitis onset, brain regions were isolated as described above for eCB analysis. Ventricular cannula placement was confirmed post-mortem with dye infusion.

### Statistical analyses

All statistics were carried out using Prism v8. For comparison of two groups, one-tailed (phenotypic confirmation of damage, corticosterone and anxiety-like behavior) or two-tailed Student’s *t* tests were used (remaining data, including correlations). For comparison of repeated measures, a repeated measure analysis of variance (ANOVA) or mixed-effect analysis was performed. For comparisons between two independent variables, two-way ANOVAs were performed. For all ANOVA analyses, significant interactions and main effects were reported, and specific group comparisons were made using Fisher’s Least Significant Difference tests. Planned comparisons based on a priori hypothesis were performed using independent *t* tests. t- or F-values, *p* values and eta squared (R^2^) are reported, as well as Pearson correlation coefficients (r) (weak = 0.1 < 0.3; moderate = 0.3 < 0.05; strong = 0.5 < 0.7; very strong = >0.7). Data are presented as mean ± standard error of the mean (SEM). Outliers were removed using the ROUT method [104], set to a 1% threshold, as previously described [83]. *p* < 0.05 was considered statistically significant. Detailed statistics for data represented in figures, as well as correlation values, are reported in Tables S2–4.

## Results

### Colitis induction produced behavioral indices of increased anxiety-like behavior

**Fig. 1:**
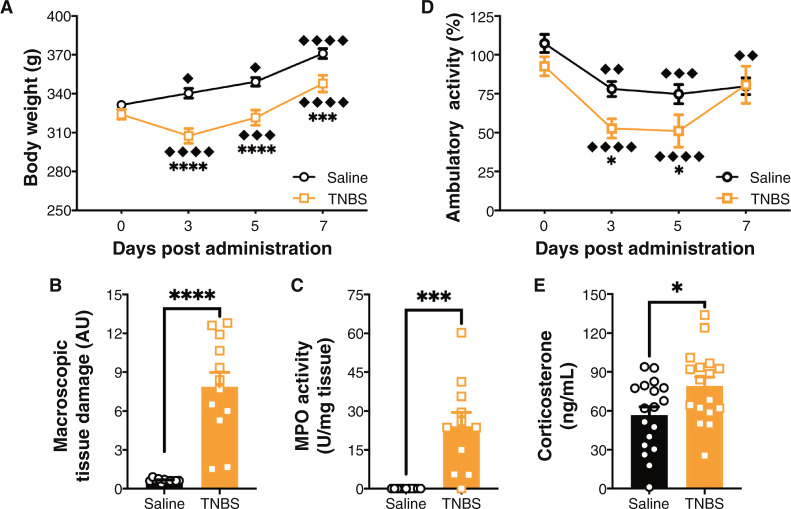
TNBS-induced colitis phenotype. **A** There was a significant interaction on body weight between time post-administration and trinitrobenzene sulfonic acid (TNBS) administration, and a main effect of both time and colitis. Saline animals gained weight each day. TNBS animals initially lost weight, but gained after Day 3. There were no differences at baseline between conditions, but there were at Days 3, 5, and 7. *n* = 12/group. ♦ *p* < 0.05, ♦♦♦ *p* < 0.001, ♦♦♦♦♦ *p* < 0.0001 compared to previously recorded weight in same condition. ***p < 0.001, ****p < 0.0001 saline vs. TNBS on the same day. **B** TNBS administration at Day 7 post-administration led to a significant increase in macroscopic tissue damage. *n* = 12/group. ****p < 0.0001 *t* test saline vs. TNBS. **C** TNBS administration at Day 7 post-administration led to a significant increase in myeloperoxidase activity (MPO). Each 1 Unit (U) of MPO activity was the amount of enzyme required to split 1µmol H_2_O_2_ per min at 25 °C. *n* = 12/group. ***p < 0.001 *t* test saline vs. TNBS. **D** There were no differences in locomotor activity between saline and TNBS groups at baseline, but there was a reduction in ambulatory activity at Day 3 and Day 5, but not at Day 7. Saline and TNBS administered animals both showed reductions in ambulatory activities compared to their baselines. *n* = 4–6/group. ♦♦ *p* < 0.01, ♦♦♦ *p* < 0.001, ♦♦♦♦ *p* < 0.0001 compared to Day 0 in same condition. *p < 0.05 saline vs. TNBS on the same day. **E** TNBS led to a significant increase in plasma corticosterone levels. *n* = 17–18/group at Day 7 post-administration. *p < 0.05 *t* test saline vs. TNBS. Saline = left, black bars with circles. TNBS = right, orange bars with squares.

Data presented on colitis phenotype (i.e., weight loss, macroscopic tissue damage and MPO activity; Fig. 1A–C) are a representative set of data from the rats used for AEA and 2-AG analysis, but these effects were consistent across all experimental cohorts. Animals administered TNBS lost weight between Day 0 and Day 3 but started gaining it again thereafter (Fig. 1A); whereas controls gained weight daily. There were no differences at baseline between saline and TNBS-treated animals, but TNBS-treated animals weighed less than saline-treated animals all other days (Fig. 1A).

Rats administered TNBS also show an increase in colonic inflammation as measured by macroscopic tissue damage (Fig. 1B) and  MPO activity (Fig. 1C) 7 days after treatment. Together these results indicate that peak weight loss occurred at Day 3, and gut inflammation was sustained at Day 7 after treatment.

Before initiating behavioral tasks, we wanted to verify that there would be no locomotor deficits (Fig. 1D), as reductions in locomotor activity can be a significant confound in behavioral tests of anxiety [105]. Using a mixed-effect model to analyze, we found that animals in both saline and TNBS groups had reduced locomotor activity after the first test day, likely due to habituation to the task. Animals administered TNBS showed reduced activity at Days 3 and 5 compared to saline-treated rats, but this difference was not present at baseline or Day 7. TNBS-treated animals also exhibited an elevation in circulating levels of corticosterone (Fig. 1E), the hormonal endpoint of the HPA axis, at Day 7. Corticosterone levels were strongly positively correlated with damage. Based on these results, we proceeded with anxiety-like behavior testing on Day 7, as at this time point animals showed no locomotor deficits, but TNBS-treated animals had sustained gut inflammation.

**Fig. 2:**
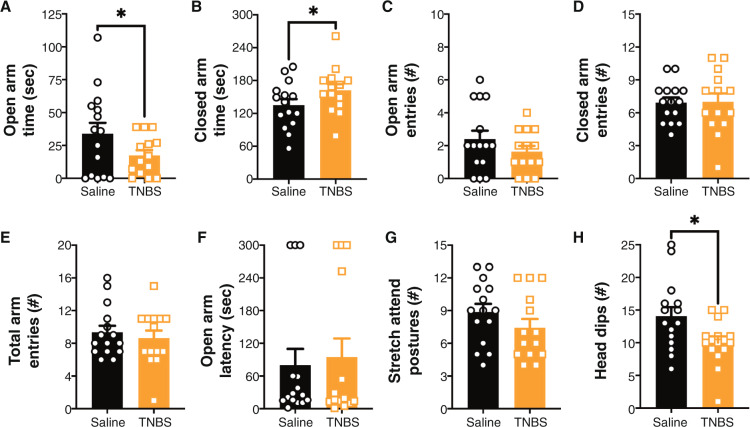
TNBS-induced colitis leads to anxiety-like behavior in the EPM at Day 7 post-administration. At Day 7 post-trinitrobenzene sulfonic acid (TNBS) administration there was an increase in anxiety-like behavior as indicated by a (**A**) reduction in time spent in the open arms, (**B**) increase in time spent in the closed arms and (H) a reduction in head dips; however, there were no effects on (**C**) open arm entries, (**D**) closed arm entries, (**E**) total arm entries, (**F**) latency to open arm or (**G**) stretch attend postures. *n* = 14–15/group. *p < 0.05 *t* test saline vs. TNBS. Saline = left, black bars with circles. TNBS = right, orange bars with squares.

In the EPM, TNBS-treated animals had increased anxiety-like behavior as indicated by a reduction in the time spent in the open arms, increased time spent in the closed arms and reduction in head dips (Fig. 2A, B, H). There were no changes in open arm entries, closed arm entries, total arm entries, latency to enter open arms, or stretch attend postures (Fig. 2C–G). There was no correlation between damage score and any of these measures.

### TNBS-induced colitis altered central endocannabinoid levels

**Fig. 3:**
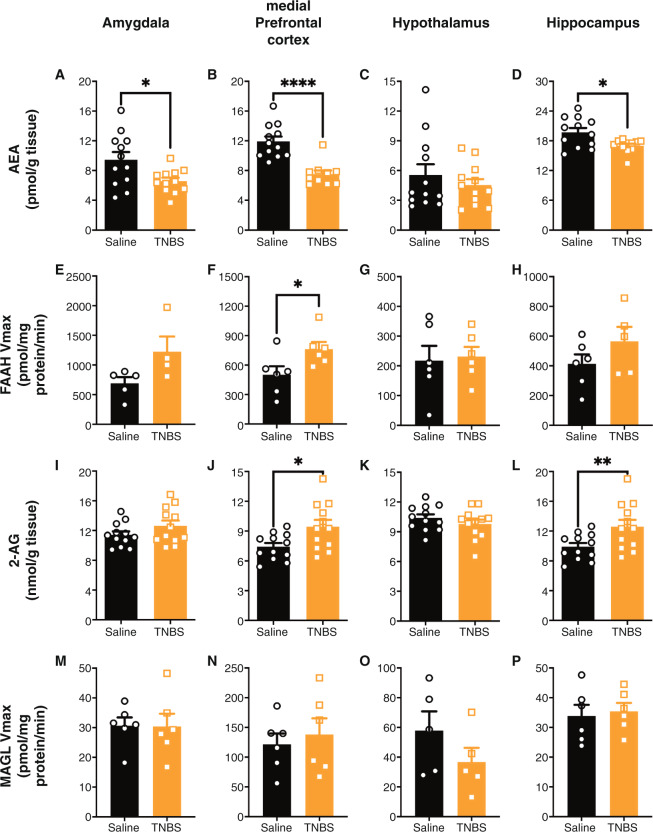
Colitis altered central endocannabinoid levels. Following trinitrobenzene sulfonic acid (TNBS) administration, at Day 7, anandamide (AEA) levels were reduced in the (**A**) amygdala, (**B**) medial prefrontal cortex and (**D**) hippocampus, but not the (**C**) hypothalamus. Concomitantly, there was an increase in AEA’s metabolic enzyme’s (fatty acid amide hydrolase (FAAH)) activity (V_max_), in the (**E**) amygdala, (**F**) medial prefrontal cortex, with no differences in the (**H**) hippocampus or (**G**) hypothalamus. In contrast to AEA levels, Day 7 2-arachindonylyl glycerol (2-AG) levels were increased in the (**J**) medial prefrontal cortex, (**L**) hippocampus, and no significant changes occurred in the (**I**) amygdala or (**K**) hypothalamus with TNBS administration. There were no differences at Day 7 post-administration in the activity of 2-AG’s metabolic enzyme (monoacylglycerol lipase (MAGL); V_max_) in the (**M**) amygdala, (**N**) medial prefrontal cortex, (**O**) hypothalamus or (**P**) hippocampus from colitis. *n* = 9–12/group for levels and *n* = 4 = 6/group for enzyme activity. *p < 0.05, **p < 0.01, ****p < 0.0001, *t* test saline vs. TNBS. Saline = left, black bars with circles. TNBS = right, orange bars with squares.

In order to investigate the role of the eCB system on colitis-induced anxiety-like behavior, we analyzed whether the eCB system was altered in Day 7 TNBS-treated rats. We found that AEA levels were reduced in the amygdala, medial prefrontal cortex and hippocampus but not in the hypothalamus in animals treated with TNBS (Fig. 3A–D). AEA levels, overall, were moderately or strongly, negatively correlated with macroscopic tissue damage, except in the hypothalamus. Concomitantly, TNBS treatment led to an increase in the hydrolytic activity of AEA’s metabolic enzyme, FAAH (V_max_), in the amygdala and medial prefrontal cortex, but not in the hypothalamus or hippocampus (Fig. 3E–H). TNBS treatment resulted in no differences in the binding affinity of AEA for FAAH (K_m_) (Table S3) in the amygdala, hypothalamus or hippocampus; however, animals with colitis had an increase in K_m_ in the medial prefrontal cortex. Similar to AEA levels, FAAH hydrolytic activity, but not binding affinity, overall, was strongly, but positively, correlated with damage score, particularly in the amygdala and medial prefrontal cortex. These data indicate that colitis is associated with an increase in corticolimbic FAAH-activity and a decline in the pool of AEA.

In contrast to AEA levels, 2-AG levels were increased in the medial prefrontal cortex and hippocampus, but not significantly changed in the amygdala or hypothalamus (Fig. 3I–L), following TNBS administration. There was no impact of TNBS-colitis on the activity of 2-AG’s metabolic enzyme (V_max_; Fig. 3M–P), MAGL, or its K_m_ (Table S3) in the amygdala, medial prefrontal cortex, hypothalamus or hippocampus. Overall, only hippocampal 2-AG was strongly, positively, correlated with damage; neither MAGL activity (excepting the hypothalamus) nor binding affinity correlated with damage score.

In addition, we examined the gene expression levels of a number of the molecular components of the eCB system (Table S4). There were no significant changes in any genes examined.

### Central FAAH inhibition reversed colitis-induced anxiety-like behavior

To determine if the elevated FAAH activity and reduced AEA levels contributed to the increase in anxiety-like behavior, we examined if acute inhibition of FAAH, to elevate AEA signaling, would counter the colitis-induced anxiety. Given that FAAH activity was broadly increased following colitis, we opted to perform a central inhibition of FAAH to determine the impact of widespread central elevations in AEA signaling. We administered a FAAH inhibitor (PF) acutely at two doses and examined anxiety-like behavior in the EPM in order to investigate the relevance of changes in eCB levels to the increase in anxiety-like behavior.

**Fig. 4:**
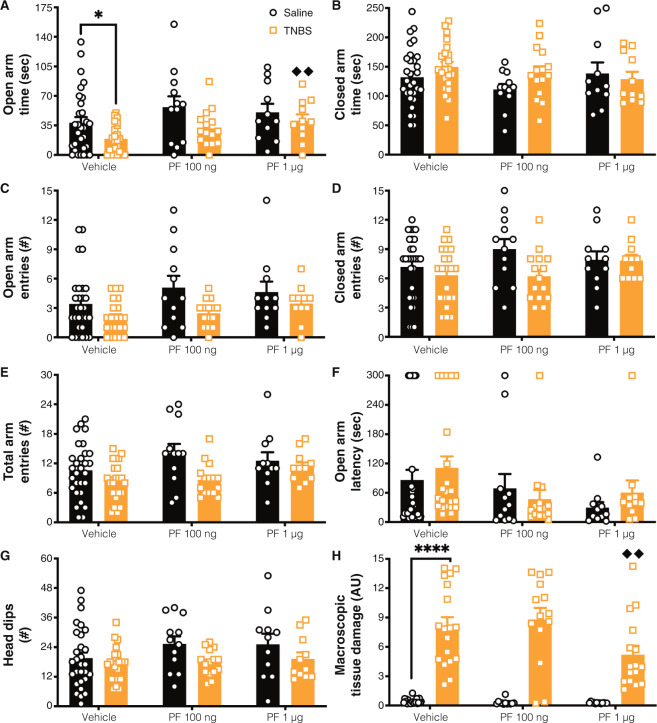
Central FAAH inhibition reversed colitis-induced anxiety-like behavior. We examined (**A**) time in the open arms and found main effects of colitis reducing open arm time, and PF administration increasing it. Planned comparisons revealed that there was a reduction in open arm time between the vehicle saline vs. TNBS groups. TNBS animals treated with 1 μg PF had significantly increased open arm time relative to vehicle treated animals. Colitis reduced (**C**) open arm entries and (**G**) head dips, but there was no effect of FAAH inhibition or interaction between colitis and PF on these measures. For (**B**) closed arm time, (**D**) closed arm entries, (**E**) total arm entries and (**F**) open arm latency there was not a significant effect of colitis, FAAH inhibition or interaction between PF and TNBS. **H** Macroscopic tissue damage was increased with colitis, but this was modulated by PF administration, specifically, with the 1 µg dose, which had reduced damage scores compared to the TNBS vehicle group. *n* = 12–24/group. *p < 0.05, ****p < 0.0001. saline vs. TNBS within same treatment, ♦♦ *p* < 0.01 vs. vehicle of same condition. Saline = left, black bars with circles. TNBS = right, orange bars with squares.

Open arm time was reduced with TNBS-induced colitis and increased with administration of the FAAH inhibitor (Fig. 4A). Based on the outcomes of the initial experiments in this study, we made the a priori hypothesis that colitis would increase anxiety-like behavior and that treatment with a FAAH inhibitor would reverse that. Analysis of these planned comparisons demonstrated that, even following cranial surgery, there was a reduction in open arm time in TNBS-treated animals treated with saline vs. control animals treated with saline, supporting the robustness of this behavioral effect (as was seen in Fig. 2A). Administration of PF dose-dependently reversed the reduction of open arm time in the EPM; whereas 100 ng PF treatment in TNBS-treated animals partially reversed the anxiety phenotype (as it was no longer significantly different relative to vehicle-control animals, but not different from TNBS-vehicle animals), TNBS-treated animals treated with 1 μg PF exhibited significantly elevated time in the open arms relative to the TNBS-vehicle treated animals (Fig. 4A). We found that TNBS-treated rats had reduced open arm entries and head dips, but neither of these were influenced by administration of PF (Fig. 4C, G). There were no significant effects on closed arm time, closed arm entries, total arm entries or open arm latency as a result of TNBS-treatment or PF administration (Fig. 4B, D–F).

Macroscopic damage of the colon was increased in TNBS-treated rats, but this was lower in the 1 µg PF dose compared to its vehicle (Fig. 4H). Specifically, there was a significant reduction in the 1 µg PF group in most scorable items, including ulceration score, diarrhea and bowel thickness (data not shown). MPO activity was also increased with TNBS-treated animals, but was not influenced by central administration of 1 µg PF (Table S2). In addition, in most indices measured, there were weak, negative correlations with damage score. Together these results indicate that central FAAH inhibition reverses colitis-induced suppression of open arm time and reduces macroscopic colonic tissue damage score.

### Central CRF-R1 signaling regulates colitis-induced alterations in AEA

As increased FAAH activity was found to contribute to the generation of colitis-induced anxiety, and given previous work that showed that activation of CRF-R1 can induce FAAH hydrolysis of AEA during psychological stress [50, 106], we examined CRF signaling as a potential upstream mechanism in our model [68].

**Fig. 5:**
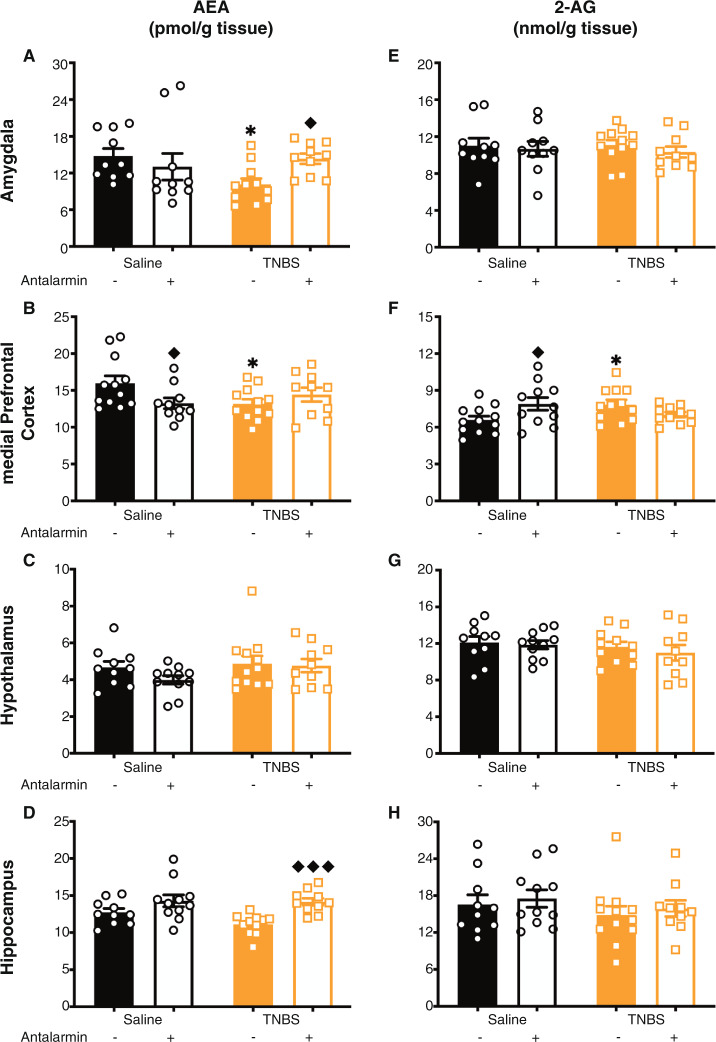
Colitis-induced reductions in AEA levels were mediated by CRF-R1. Antalarmin (an antagonist for the corticotropin releasing factor receptor type 1 (CRF-R1)) reversed the colitis-induced reduction in anandamide (AEA) levels in the (**A**) amygdala and (**D**) hippocampus. In the (**C**) hypothalamus, there was no TNBS effect (as in Fig. 3C), and no effect of antalarmin. In the (**B**) medial prefrontal cortex, there was an interesting effect where antalarmin in the saline animals reduced AEA levels, but did not alter the TNBS-induced reduction of AEA levels. However, antalarmin had no effect on 2-arachidonylglycerol (2-AG) levels in the (**E**) amygdala, (**G**) hypothalamus or (**H**) hippocampus; however, in the (**F**) medial prefrontal cortex, antalarmin increased 2-AG levels in saline animals, but did not alter 2-AG levels in TNBS animals. *n* = 10–12/group. *p < 0.05 saline vs. TNBS of same treatment, ♦ *p* < 0.05, ♦♦♦ *p* < 0.001 vs. vehicle of same condition. Saline = left, black bars with circles. TNBS = right, orange bars with squares. Vehicle = left of each pair, filled bars. Antalarmin = right of each pair, open bars.

We investigated if blocking central CRF-R1 with an antagonist, antalarmin, for the 7-days post-TNBS administration altered colitis-induced changes in AEA levels. While this caused no changes to TNBS-induced increases in macroscopic tissue damage (Table S2), antalarmin reversed colitis-induced reductions in AEA levels (Fig. 5A, D) in the amygdala and hippocampus. As in Fig. 3C, there was no effect of TNBS on hypothalamic AEA levels, nor was there an effect of antalarmin (Fig. 5C). In the medial prefrontal cortex, antalarmin alone reduced AEA levels, but did not alter TNBS-induced changes in AEA levels (Fig. 5B). CRF-R1 antagonism had no effect on 2-AG levels in the amygdala, hypothalamus and hippocampus (Fig. 5E, G, H). However, in the medial prefrontal cortex (Fig. 5F), antalarmin administration in saline animals increased 2-AG but did not alter 2-AG levels in the TNBS-treated animals. No overall pattern emerged with regards to correlation with macroscopic damage. Together these data demonstrated that the colitis-induced reductions in AEA, at least within the amygdala and hippocampus, were driven through CRF-R1 signaling.

### TNBS-colitis also reduced central AEA levels in female rats

Comorbid anxiety with IBD is not restricted to males and is also observed in females [16, 27]. To this end, we examined if there was a similar alteration in the eCB system as a result of TNBS administration in female rats in order to understand potential generalizability across sexes of the phenomenon we have demonstrated. Female rats also showed an increase in macroscopic tissue damage and MPO activity. Similar to male rats, there was a reduction (although smaller) of AEA levels in the medial prefrontal cortex (Fig. S1B) and no change in the hypothalamus (Fig. S1C), but reductions seen in males in the amygdala and hippocampus (*p* > 0.05) (Fig. S1AD) were not seen. Also, in contrast to male levels, there were no alterations (*p* > 0.05) in 2-AG levels in any of these areas (Fig. S1E–H). Unlike what was seen in males, neither AEA nor 2-AG levels were correlated with macroscopic damage.

## Discussion

We demonstrate that the TNBS-model of colitis in rats, consistent with other rodent models of colitis [70–73, 107–109], produces an increase of anxiety-like behavior (Fig. 2), similar to the well-established comorbidity of colitis and anxiety in humans [1–5, 22, 27]. Colitis also resulted in an increase in FAAH-mediated hydrolysis of AEA across corticolimbic structures (Fig. 3) important for the regulation of affective behavior [105]. The magnitude of reductions in AEA and increases in FAAH activity is overall correlated with macroscopic damage, suggesting that the greater the disease severity the larger the impact on FAAH and AEA. This reduction in AEA signaling was mediated by central CRF-R1 (Fig. 5), and it contributed to the development of colitis-induced anxiety, as this was reversed by central inhibition of FAAH (Fig. 4). Together these data indicate that sustained peripheral inflammation can modulate affective behavior through an attenuation of central AEA signaling, which is driven by a recruitment of stress-responsive signaling systems. As such, this would suggest that inhibition of FAAH could represent a novel therapeutic approach to managing comorbid anxiety in peripheral inflammatory diseases.

Endocannabinoid signaling is well established to regulate affective behavioral processes such as anxiety through actions localized within the amygdala, medial prefrontal cortex and hippocampus [43, 110]. The current data extend these findings to demonstrate that a sustained inflammatory state results in a loss of central AEA signaling that contributes to the development of anxiety. Previous work has suggested that AEA and FAAH may be involved in behavioral changes produced by inflammation. For example, administration of the viral mimetic poly I:C produces changes in thermoregulation, pain sensitivity and anxiety, which are reversed by administration of a FAAH inhibitor [111]. In addition, acute early life inflammatory events have been shown to reduce social behavior during adolescence, a process that is also reversible through pharmacological inhibition of FAAH [112]. The current data, however, are the first demonstration that a sustained peripheral inflammatory insult reduces AEA levels and increases FAAH activity via central CRF-R1 activity, to increase anxiety, and thereby provides a putative model by which peripheral inflammation can modulate the central regulation of affective behavior.

Recent work from our group shows that both male and female mice exhibit anxiety-like behavior in a dextran sulfate sodium model of colitis [73]. Here we show in males that anxiety-like behavior induced by TNBS colitis is mediated through an CRF-R1 suppression of AEA levels. We also demonstrate in female rats that TNBS administration leads to a reduction of AEA levels, albeit to a lesser magnitude than in the males. It is possible that in females this reduction of AEA levels also contributes to the anxiety like-behavior observed across models, as previous work has demonstrated a correlation between AEA levels and anxiety-like behavior [113]; and, the difference in magnitude of AEA changes between sexes may contribute to the sex differences in anxiety-like behavior previously observed [73].

The finding that CRF-R1 activity mediates the colitis-induced reduction in AEA content broadens previous work indicating that CRF and FAAH exhibit an intricate relationship in the regulation of affective behavior [50, 106]. Chronic exposure to glucocorticoids results in sustained elevations in central FAAH hydrolysis and this is mediated by the elevated CRF/CRF-R1 activity as this effect of glucocorticoids is blocked by continuous administration of a CRF-R1 antagonist and is replicated by genetic overexpression of forebrain *Crh* [50, 106]. Inflammation is well-established to increase drive on the HPA axis, likely in an auto-regulatory manner where the elevations in potent anti-inflammatory glucocorticoids act to dampen inflammation itself [35, 114, 115]. Consistent with this, our data replicate previous studies [116, 117] showing that TNBS-colitis results in chronic elevations in corticosterone secretion, which is in line with the established increase in central *Crh* expression in rodent models of gut inflammation [54, 57, 59, 66, 67, 69, 89]. These data would suggest colitis-induced inflammation produces sustained adrenocortical responses, which result in the upregulation of CRF levels in the brain, producing an increase in FAAH activity and a reduction in AEA signaling. Consistent with previous work [50], this effect of glucocorticoids and CRF-R1 signaling on FAAH does not appear to be mediated by transcriptional changes in gene expression.

An unexpected finding of this study was that acute central inhibition of FAAH reduced the severity of colitis. Endocannabinoids are well-established anti-inflammatory molecules [118, 119], and FAAH inhibitors have been repeatedly found to be capable of reducing multiple aspects of gut inflammation across several animal models [89–91, 96, 120, 121]. While these anti-inflammatory effects of AEA signaling in colitis are largely due to peripheral actions on colonic tissue directly or local immune cells, there is evidence that central cannabinoid type 1 receptors (CB1) contribute to reducing inflammation in colitis [122]. It is also possible that PF entered the circulation, elevating AEA levels outside of the brain to influence the damage score. That said, the magnitude of reduction of colitis damage in the current study from central FAAH inhibition was relatively minor. Regardless, these data support previous findings that central FAAH inhibition is capable of modulating colonic inflammation.

In addition to reduced AEA, we also found that colitis was associated with elevations in 2-AG throughout several corticolimbic structures (Fig. 2). Prolonged elevations in CRF signaling have been found to produce elevations in tissue 2-AG levels [106]. This effect was not as robust as the reduction in AEA, as it was largely lost following cannulation surgery, and not seen in females. Unlike the reductions in AEA, the relevance of these increases in 2-AG during colitis has yet to be elucidated. As stress-induced elevations in 2-AG signaling have been proposed to produce both anxiolytic and anxiogenic effects [43], future work is required to examine this question in more depth.

We did not investigate which receptors mediate the anxiolytic effect observed herein. Previous work with psychological stress points to a role for CB1 in this regard [43]. AEA’s anxiolytic effect seems to be due to its signaling at the CB1 receptor [43]. Furthermore, differently from AEA, the ability of 2-AG to buffer anxiety is linked to signaling at the CB1 receptor, but also the CB2 receptor [123–126]. Given our anxiolytic effect was observed with FAAH inhibition, which elevates AEA and not 2-AG, it was likely through a CB1 mechanism; however, future work will have to elucidate these specifics, especially as AEA can also act on the transient receptor potential cation channel subfamily V member 1 (TRPV1) and peroxisome proliferator-activated receptors (PPARs). More so, FAAH also metabolizes oleoylethanolamide (OEA) and palmitoylethanolamide (PEA) which have also been implicated in anxiety and inflammation [127–133].

Together, these data demonstrate that the induction of colitis results in a suppression of central AEA signaling via a CRF-R1 mediated increase in FAAH activity, which then promotes the development of anxiety. Given the similarities seen to chronic stress and glucocorticoid exposure [50, 106], this suggests that compromised central AEA signaling may be a broad mechanism favoring the development of anxiety in response to a host of psychological or physiological insults, particularly those that produce increased demand on the HPA axis. As such, these data would support the investigation of FAAH inhibitors as a treatment approach in chronic inflammatory disease states, both for the inflammatory pathology itself but also the psychiatric comorbidities. FAAH inhibitors have already been established in humans to reduce anxiety that develops during cannabis withdrawal [134], dampen the subjective and physiological responses to stress [135] and produce clinically relevant anxiolysis in social anxiety disorder [136], indicating their feasibility and potential efficacy for the management of affective disturbances in humans. In line with this, many individuals with chronic inflammatory diseases use cannabis which is associated with broad improvements in affective state and quality of life [137, 138], suggesting that cannabinoids may also have some therapeutic value in this domain. Therefore, there is potential for FAAH inhibition on both the primary outcomes of inflammatory diseases, as well as comorbid psychiatric issues and quality of life measures, serving as a dual-pronged therapeutic.

## Funding and disclosures

This work was supported by grants from the Canadian Institutes of Health Research (CIHR; FDN333950-MNH; FDN148380-KAS; PJT159454-QJP, MH, KAS); Alberta Innovates Health Solutions (AIHS) CRIO Project 201200828-QJP, KAS. HAV received stipend funding from CIHR (Vanier CGS), University of Calgary (UofC; Killam Pre-doctoral Laureate), AIHS and Branch Out Neurological Foundation (BONF); MM received fellowship support from AIHS and CIHR; VC received a studentship from UofC; KT received studentships from the National Sciences and Engineering Research Council (NSERC) and AIHS; KL received a studentship from BONF; AS received salary support from the UofC Mathison Centre for Mental Health Research and Education. MNH is the recipient of a Tier II Canada Research Chair. KAS holds the Crohn’s and Colitis Canada Chair in IBD Research at the UofC. Funding agencies had no influence on the design, execution or publishing of this work. MNH is a scientific advisor for Sophren Therapeutics and Lundbeck. All other authors have no disclosures to report.

## Supplementary information

Supplemental

Supplemental Table 1

Supplemental Table 2

Supplemental Table 3

Supplemental Table 4

Supplemental Figure 1
