# Supplementary material for: Comorbid anxiety-like behavior in a rat model of colitis is mediated by an upregulation of corticolimbic fatty acid amide hydrolase
Source: Neuropsychopharmacology. 2021 Jan 15;46(5):992–1003. doi: 10.1038/s41386-020-00939-7 (PMC8115350; doi:10.1038/s41386-020-00939-7)
Supplement: Supplementary file 1 — Supplemental [file 41386_2020_939_MOESM1_ESM.docx]

**Supplemental Material**

**Materials and Methods**

Macroscopic Tissue Damage Score

Upon removal, colons were washed with ice-cold physiological saline (0.9%) and cut open longitudinally and macroscopically scored for damage and inflammation, based on the presence of adhesions, diarrhea and degree of ulceration. This score was adapted from those previously reported (1–4). Specifically the score is the summation of: ulceration (0—normal appearance; 1—focal hyperemia, no ulcers; 2—ulceration without hyperemia or bowel wall thickening; 3—ulceration with inflammation at one site; 4—2 or more sites of damage and ulceration; 5—major sites of damage extending >1 cm along length of colon; 6+—major sites of damage extending >2 cm along length of colon, increased by 1 for each 1 cm of damage); + adhesions (0—no adhesions; 1—minor adhesions (e.g. one organ involved; easily separated from other tissues); 2—major adhesions); + diarrhea (0—absent; 1—present); + thickness (maximal bowel thickness in mm).

Myeloperoxidase Activity

After it was scored for macroscopic damage, a <100mg sample of colon was excised, snap frozen and stored at -80°C for later use in a myeloperoxidase (MPO) activity assay, which is a marker of neutrophil mediated inflammation. Adapted from previous work (1–4), frozen colons were weighed, and placed in a solution of hexadecyltrimethylammonium bromide (HTAB) (Sigma-Aldrich, Darmstadt, Germany, #H5882) in potassium phosphate (PP) buffer (95:5 5% monobasic potassium phosphate:5% dibasic potassium phosphate) (50mM, pH 6.0, 0.5% HTAB weight/volume; 1mL buffer/50mg tissue). Homogenization of the colon occurred using 50mm stainless steel beads (Qiagen, Hilden, Germany, #69989) and the TissueLyser LT bead homogenizer (Qiagen) for 10 minutes (min) at 50Hz. Samples were then centrifuged for 10min at 15871 x *g* at 4°C. 7µL of the supernatant was added to 200µL of 0.00005% H_2_O_2_ in o-dianisidine dihydrochloride (Sigma-Aldrich, #D3252) buffer (per 150mL: 16.7 mg o-dianisidine dihydrochloride, 90mL ddH_2_O, 10mL PP buffer, 50µL 1% H_2_O_2_) in a 96 well-plate. Absorbance (A) was measured every 30 seconds (sec) at 450nm over 1 min (3 reads total). Each 1 Unit (U) of MPO activity was the amount of enzyme required to split 1µmol H_2_O_2_ per min at 25°C.

Elevated Plus Maze (EPM)

The EPM (Med Associates, Fairfax, VT, USA) was made of black plastic, elevated 48cm above the floor, with open arms that were 48cm long and 15cm wide, with closed arms having the same width and length and a wall height of 38cm. The open arms were exposed to 50 lumens of light, and the closed arms were at 5-10 lumens of light; the maze was under-lit with infrared light to facilitate video-recording with an infrared sensing camera (5). Each session of the EPM lasted for 5 min and began when the rats were placed in the center of the maze facing an open arm. Total time spent in open and closed arms, latency to enter an open arm, total number of entries into the open and closed arms, stretch attend postures (rat stretches forward then returns to the original position without moving forward; associated with increased anxiety-like behavior and risk assessment behavior) and head dips (rat leaned head over side of EPM investigate below EPM; associated with reduced anxiety-like behavior) were scored by a blinded observer. Entries and time spent in the open/closed arms were only considered if all 4 paws and full body not including the tail were into the arms (5). The EPM was thoroughly cleaned between each test run.

Endocannabinoid Measurement

AEA and 2-AG were measured using liquid chromatography/tandem mass spectrometry (6). Dissections and excisions of corticolimbic brain structures were performed on ice and samples were then immediately snap frozen and stored at -80°C as previously extensively described (7). Frozen tissue was homogenized in a borosilicate glass tube containing 2mL of acetonitrile with 5pmol d8-AEA (Cayman Chemical Company, Ann Arbor, Michigan, USA, #390050) and 5nmol d8-2-AG (Cayman Chemical Company, #362160) with a glass rod. Following sonication of the tissue, an overnight incubation at -20°C and centrifugation at 1,500 x *g*, proteins were removed. Supernatants containing lipids were transferred to a new tube and evaporated under nitrogen gas, washed with acetonitrile and evaporated again. Final reconstitution was in 200µL of acetonitrile before storage at -80°C.

Analysis of AEA and 2-AG were conducted through liquid chromatography/tandem mass spectrometry on a Eksigent ekspert micro liquid chromatographer 200 coupled to an AB Sciex Qtrap 5500 mass spectrometer (SCIEX, Framingham, MA, USA), which was outfitted with a Turbo V Spray ion source at the Southern Alberta Mass Spectrometry Centre at the University of Calgary (6). The separation of AEA and 2-AG occurred using an Eksigent HALO C18 high performance liquid chromatography column (1 x 50mm, 2.7µm particle size, 90A pores) (SCIEX), with a fast, 5 min gradient, at a flow rate of 30µL/min, with a solvent consisting of 15% of mobile phase A (10mM ammonium acetate in water) and 85% mobile phase B (acetonitrile). AEA (Cayman Chemical Company, #90050) and AEA-d8 were monitored in their protonated form (348m/z and 356m/z, respectively). 2-AG (Cayman Chemical Company, #62160) and 2-AG-d8 were monitored as ammonium ion adducts (396 m/z and 404 m/z, respectively). Cannabinoids were detected in multiple reaction monitoring mode using Q1 and Q3 at unit resolution and collision energies optimized for these transitions: AEA (348>62), AEA-d8 (356>62), 2-AG (396>287 and 396>259) and 2-AG-d8 (404>294 and 404>313). Quantification was based on extracted ion chromatogram peak area (however, since 2-AG isomerizes to 1-AG during sample preparation, the peak of both isomers was used for quantification). Data were processed using Analyst 1.5.2 software (SCIEX) and linear regression of the ratio of analyte peak areas to internal standard was performed over analyte concentration range. For this method, a calibration set of six dilutions of both AEA (0.05-50nM) and 2-AG (0.025-25µM) was utilized.

Enzyme Activity Assays

Corticolimbic brain structures were excised on ice and samples were then immediately snap frozen and stored at -80°C. Prior to enzyme activity assays, corticolimbic brain regions were homogenized and membrane fractions were isolated as described previously (8). Briefly, tissue was homogenized in 10x its mass in volume of tris-magnesium-ethylenediaminetetraacetic acid (EDTA) (TME) Buffer (50mM Tris HCl, 1mM EDTA and 3mM MgCl_2_, pH 7.4) using a glass mortar and pestle. Homogenized tissue was centrifuged for 20 min at 18,000 x *g* at 4°C. Pellets containing crude membrane fractions were resuspended again in 10x volume. A Pierce bicinchoninic acid (BCA) protein assay was performed to quantify protein concentration according the manufacturer’s protocol (Thermo Fisher Scientific, Waltham, MA, USA, #23225) (9).

FAAH activity was measured as the conversion of [^3^H]-AEA (American Radiolabeled Chemicals, Inc., St. Louis, MO, USA, #ART 0626) to [^3^H]-ethanolamine as previously described (10). Briefly, in triplicate, membrane fractions (10µg) were incubated in TME Buffer with bovine serum albumin (BSA) (Millipore Sigma, Darmstadt, Germany, #Sigma A6003), 0.2nm [^3^H]-AEA and non-tritiated AEA (Cayman Chemical Company, #90050) in 8 concentrations (10nM to 10mM). Reactions were carried out at 30°C for 30 min and stopped by addition of 2mL of chloroform/methanol (1:2). Reacted products then sat at room temperature (RT) for 30 min, with intermittent vortexing, after which 0.67mL of chloroform and 0.6mL of water were added. Organic and aqueous phases were separated via centrifugation (1500 x *g* for 10 min at 4°C). The amount of [^3^H] in 0.5mL of the aqueous phase was obtained using liquid scintillation counting in disintegrations per min (DPM). The conversion of [^3^H]-AEA to [^3^H]-ethanolamine was calculated. As the radioactive epitope was present on the ethanolamine portion, radioactivity present in the aqueous phase (containing [^3^H]-ethanolamine but not [^3^H]-AEA) represented the amount of metabolized AEA. The maximal hydrolytic activity of FAAH (V_max_) and the binding affinity of AEA for FAAH (K_m_) for this conversion were determined by fitting the data to the Michaelis-Menten equation using Prism v8 (GraphPad, San Diego, CA, USA, RRID:SCR_002798).

Similarly, MAGL activity was measured as the conversion of [^3^H]-2-oleoylglycerol (2-OG) (American Radiolabeled Chemicals, Inc., #ART 0400) to [^3^H]-glycerol, as previous described (11). Briefly, in triplicate, membranes (at a concentration of 300µg/mL) were incubated in 0.5mL of TME buffer containing 1mg/mL BSA, 300nM URB597 (a FAAH inhibitor) (Cayman Chemical Company, #10046) and 100,00 DPM of [^3^H]-2-OG. Non-tritiated 2-OG (Sigma-Aldrich, #M2787) was added in 6 concentrations between 10 and 500µM. Similar to the FAAH enzymatic activity assay, reactions were carried out at 30°C for 30 min and stopped by addition of 2mL of chloroform/methanol (1:2). Reacted products then sat at RT for 30 min, with intermittent vortexing, after which 0.67mL of chloroform and 0.6mL of water were added. Organic and aqueous phases were separated via centrifugation (1500 x *g* for 10 min at 4°C). The amount of [^3^H] in 0.5mL of the aqueous phase was obtained using liquid scintillation counting in DPM. The conversion of [^3^H]-2-OG to [^3^H]-glycerol was calculated. Similar to the FAAH assay as described above, only radioactivity attached to glycerol, not that attached to 2-OG, is measured, and thus representative of metabolized 2-OG. The maximal hydrolytic activity of MAGL (V_max_) and the binding affinity of 2-OG for MAGL (K_m_) for this conversion were determined by fitting the data to the Michaelis-Menten equation using Prism v8.

Gene Expression Analysis

Messenger ribonucleic acid (mRNA) isolation and complementary deoxyribonucleic acid (cDNA) synthesis from corticolimbic brain regions was carried out as previously described (8,9,12) using a Qiagen RNeasy Plus Universal Mini kit (Qiagen, #73404). Brain regions were homogenized using 50mm stainless steel beads (Qiagen, #69989) and the TissueLyser LT bead lyser (Qiagen) (50 Hz for 2 min) in 1mL of QIAzol lysis reagent. Samples were homogenized, then incubated with genomic DNA (gDNA) eliminator solution and chloroform and spun at 12,000 x *g* for 15 min at 4°C. Total mRNA was isolated from the aqueous phase of each sample using a Qiagen RNeasy Plus Universal Mini kit with a Qiacube according to the manufacturer’s protocol. Samples were eluted in 100µL water and mRNA content and purity were ascertained using a Nanodrop 2000 spectrophotometer (Thermo Fisher Scientific, NC-2000; RRID:SCR_018042) (A260 nm/A280 nm ratio) before they were aliquoted and frozen at -80°C.

mRNA was transformed into cDNA using the QuantiTect Reverse Transcription Kit (Qiagen, #205314) according to the manufacturer’s protocol. 2µg of mRNA per sample was incubated in gDNA Wipeout Buffer for 2 min at 42°C, after which, samples were incubated with buffer containing RNase inhibitors, a reverse transcriptase enzyme, a random primer mix and (deoxynucleoside triphosphate) dNTPs for 15 min at 42°C, which was followed by an inactivation step at 95°C for 3 min. cDNA quantification was determined using a Nanodrop spectrophotometer, and then samples were aliquoted and frozen at -80°C. cDNA aliquots were diluted in water to a final cDNA concentration of 25ng/µL.

Primers were designed using PrimerQuest software (IDTDNA, Coralville, IO, USA) (8,9,13,14). All efforts were taken to check literature for duplication of previous primers, and any replication of previously published primers not cited is purely accidental and likely was due to use of the same software program. mRNA sequences of primers of interest were obtained by searching the NCBI Nucleotide database (RRID:SCR_006472). PrimerQuest was instructed to create primers that would result in primer products that were not larger than 200 base pairs (bp) and had annealing temperatures of 55°C ± 1°C. Primer pairs that met these criteria were analyzed with the NCBI BLAST tool (RRID:SCR_004870), to check for specificity, as well as dimerization and hairpin probability. Primers were diluted in RNase-free water, for a final stock concentration of 10µM. For a list of primer sequences see Supplemental Table 1.

Quantitative polymerase chain reaction (qPCR) was performed as previously described (8,9,12) using PerfeCTa SYBR Green Fast Mix (QuantaBio, Beverly, MA, USA, #95072) on a RotoGene Q light cycler (Qiagen) according to the manufacturer’s instructions. Briefly, 50ng of cDNA, primers (1µM final concentration) and buffer containing SYBR Green and dNTPs were added to tubes for a final volume of 10µL.

Each primer was analyzed in a serially diluted standard curve in triplicate to assess proper reaction conditions (i.e. number of cycles and annealing temperature) and check primer reaction efficiency (must be between 80% and 120%). All reactions were run with the following cycler specifications: 3 min at 90°C, followed by 40 cycles of 90°C for 10 seconds (sec) and 60°C for 30 sec. A melt step was performed after each run to assess for single products. Each primer for genes of interest was compared against all primers for reference genes to ascertain which one had similar reaction efficiencies. For each gene of interest, one reference gene was chosen that matched its efficiency.

As all samples per brain region could not fit on one run, each machine run contained at least one sample from each group. For each run, a standard curve for each primer, both the primer for the reference gene and gene of interest, was performed, to validate primer efficiency across multiple runs. For each sample, reference genes were assayed in triplicate, genes of interest were assayed in triplicate, and a no template control was assayed.

Using the RotoGene Q software, the C_t_ value, or the point at which fluorescent detection crosses the threshold, was assayed for each well. The triplicate C_t_ value per sample was averaged for both the reference gene and gene of interest. The delta C_t_ between the reference gene and gene of interest was calculated and then the delta, delta C_t_, which was the change between the control (saline) and test (TNBS) groups was calculated. Data were normalized so that the average of the control group was 1.

**References**

1. McCafferty DM, Sharkey KA, Wallace JL (1994): Beneficial effects of local or systemic lidocaine in experimental colitis. *Am J Physiol* 266: G560-567.

2. Natah SS, Mouihate A, Pittman QJ, Sharkey KA (2005): Disruption of the blood-brain barrier during TNBS colitis. *Neurogastroenterol Motil* 17: 433–446.

3. Spencer SJ, Hyland NP, Sharkey KA, Pittman QJ (2007): Neonatal immune challenge exacerbates experimental colitis in adult rats: potential role for TNF-alpha. *Am J Physiol Regul Integr Comp Physiol* 292: R308-315.

4. Riazi K, Galic MA, Kuzmiski JB, Ho W, Sharkey KA, Pittman QJ (2008): Microglial activation and TNFalpha production mediate altered CNS excitability following peripheral inflammation. *Proc Natl Acad Sci USA* 105: 17151–17156.

5. Morena M, Aukema RJ, Leitl KD, Rashid AJ, Vecchiarelli HA, Josselyn SA, Hill MN (2019): Upregulation of Anandamide Hydrolysis in the Basolateral Complex of Amygdala Reduces Fear Memory Expression and Indices of Stress and Anxiety. *J Neurosci* 39: 1275–1292.

6. Qi M, Morena M, Vecchiarelli HA, Hill MN, Schriemer DC (2015): A robust capillary liquid chromatography/tandem mass spectrometry method for quantitation of neuromodulatory endocannabinoids. *Rapid Commun Mass Spectrom* 29: 1889–1897.

7. Hill MN, Karatsoreos IN, Hillard CJ, McEwen BS (2010): Rapid elevations in limbic endocannabinoid content by glucocorticoid hormones in vivo. *Psychoneuroendocrinology* 35: 1333–1338.

8. Gray JM, Vecchiarelli HA, Morena M, Lee TTY, Hermanson DJ, Kim AB, *et al.* (2015): Corticotropin-releasing hormone drives anandamide hydrolysis in the amygdala to promote anxiety. *J Neurosci* 35: 3879–3892.

9. Vecchiarelli HA, Gandhi CP, Gray JM, Morena M, Hassan KI, Hill MN (2016): Divergent responses of inflammatory mediators within the amygdala and medial prefrontal cortex to acute psychological stress. *Brain Behav Immun* 51: 70–91.

10. Atsak P, Morena M, Schoenmaker C, Tabak E, Oomen CA, Jamil S, *et al.* (2018): Glucocorticoid-endocannabinoid uncoupling mediates fear suppression deficits after early - Life stress. *Psychoneuroendocrinology* 91: 41–49.

11. Berger AL, Henricks AM, Lugo JM, Wright HR, Warrick CR, Sticht MA, *et al.* (2018): The Lateral Habenula Directs Coping Styles Under Conditions of Stress via Recruitment of the Endocannabinoid System. *Biol Psychiatry* 84: 611–623.

12. Vecchiarelli HA, Gandhi CP, Hill MN (2016): Acute Psychological Stress Modulates the Expression of Enzymes Involved in the Kynurenine Pathway throughout Corticolimbic Circuits in Adult Male Rats. *Neural Plast* 2016: 7215684.

13. Wu SW, Lindberg JE, Peters JH (2016): Genetic and pharmacological evidence for low-abundance TRPV3 expression in primary vagal afferent neurons. *Am J Physiol Regul Integr Comp Physiol* 310: R794-805.

14. Nakatsu Y, Nakagawa F, Higashi S, Ohsumi T, Shiiba S, Watanabe S, Takeuchi H (2018): Effect of acetaminophen on osteoblastic differentiation and migration of MC3T3-E1 cells. *Pharmacol Rep* 70: 29–36.
