# Supplementary material for: Comorbid anxiety-like behavior in a rat model of colitis is mediated by an upregulation of corticolimbic fatty acid amide hydrolase
Source: Neuropsychopharmacology. 2021 Jan 15;46(5):992–1003. doi: 10.1038/s41386-020-00939-7 (PMC8115350; doi:10.1038/s41386-020-00939-7)
Supplement: Supplementary file 2 — Supplemental Table 1 [file 41386_2020_939_MOESM2_ESM.docx]

**Supplemental Table 1. qPCR Primer List**

| **Gene** | **Protein** | | **Forward** | **Reverse** |
| --- | --- | --- | --- | --- |
|  | |  | | |
| *Reference Genes* | | | | |
| *B2m*^(9)^ | β_2_ microglobulin | | CAGTTCCACCCACCTCAAATAG | GTGTGAGCCAGGATGTAGAAAG |
| *Rplp0*^(9)^ | Ribosomal protein P0 (RPLP0) | | AAGGTCGAAGCAAAGGAAGAG | TTAAGCAGGCTGACTTGGTG |
| *Rplp1*^(9)^ | Ribosomal protein P1 (RPLP1) | | CAGTCCACAACATGGCTTCT | CATTGATCTTATCCTCCGTGACC |
| *Rlp2*^(8)^ | Ribosomal protein P2 (RPLP2) | | CGCTACGTTGCCTCTTATCT | GCCCACGCTGTCTAGTATTT |
| *Tbp*^(9)^ | TATA box binding protein | | TCATGGTGCGTGACGATAAC | CTGGTCCATGACTCTCACTTTC |
| *Genes of Interest (Endocannabinoid)* | | | | |
| *Abhd4* | α,β-hydrolase 4 (ABHD4) | | TCCAACCCAGTTTCCACTTAC | GTGTTATATCCTGCCCTGATCTC |
| *Abhd6* | α,β-hydrolase 6 (ABHD6) | | GGTCAAGTTCCTTCCCAAGAA | CAACTATGGACAGGTCATCCAG |
| *Abhd12* | α,β-hydrolase 12 (ABHD12) | | GCCACCAGATGCCCTTATATT | CAGTCAAAGCCTGGGAAGTATC |
| *Cnr1* | Cannabinoid receptor 1 (CB1) | | GTAAGAGGTGCTGGAAAGTAGAG | GGTTGAAGAAGGCCGTAGAA |
| *Cnr2* | Cannabinoid receptor 2 (CB2) | | TGCTACCCACCTACCTACAA | GAACAGGGACTAGGACAACAAG |
| *Dagla* | Diacylglycerol lipase (DAGL)α | | TCGGTAACAGGGAGGAGATT | GTGCCATACCAGAACAGAGATAC |
| *Daglb* | Diacylglycerol lipase (DAGL)β | | TATATCCGACTGGCCCTCTT | ACTGTCCTGTCACATTGGATAC |
| *Faah*^(8)^ | Fatty acid amide hydrolase (FAAH) | | GGAGCTAAGGAGTGAGTATTTC | CACCTGTCCTTATCCCATTAC |
| *Fabp7* | Fatty acid binding protein (FABP)7 | | CGTTGCTGTTCGCTGTTATG | CAGTGCTTCAGTAGCTGGATAA |
| *Magl* | Monoacylglycerol lipase (MAGL) | | GAGCTAGGTGTCTACCCTGAAT | GCACTAGGGTACTAGGGTTTCT |
| *Napepld* | *N*-acyl phosphatidylethanolamine-specific phospholipase D (NAPE-PLD) | | GGAGGAGGACGTAACCAAATC | ATCTGAGCACGTTTGGGATAG |
| *Trvp1*^(13,14)^ | transient receptor potential cation channel subfamily V member 1 (TrpV1) | | GGCTGTCTTCATCATCCTGTTA | GTTCTTGCTCTCTTGTGCAATC |
