# Supplementary material for: Comorbid anxiety-like behavior in a rat model of colitis is mediated by an upregulation of corticolimbic fatty acid amide hydrolase
Source: Neuropsychopharmacology. 2021 Jan 15;46(5):992–1003. doi: 10.1038/s41386-020-00939-7 (PMC8115350; doi:10.1038/s41386-020-00939-7)
Supplement: Supplementary file 4 — Supplemental Table 3 [file 41386_2020_939_MOESM4_ESM.docx]

**Supplemental Table 3. Effects of Colitis on AEA’s and 2-AG’s Binding Affinity for FAAH and MAGL**

| **Region** | **Saline** | **TNBS** | **Statistics** |
| --- | --- | --- | --- |
|  |  |  |  |
| *FAAH K_m_* | | | |
| Amygdala | 0.44±0.09, n=5 | 0.39±0.05, n=4 | t(7)=0.50; p=0.63; R^2^=0.03 |
| *Correlation with Damage Score* | |  |  |
| r=-0.13, p=0.75 | *r=0.91, p=0.03* | r=0.30, p=0.70 |  |
| medial Prefrontal Cortex | 0.41±0.07, n=6 | 0.64±0.08, n=6 | ** t(10)=2.26; p=0.05; R^2^=0.34* |
| *Correlation with Damage Score* | |  |  |
| *r=0.90, p<0.0001* | r=0.51, p=0.30 | r=0.02, p=0.96 |  |
| Hypothalamus | 0.67±0.24, n=6 | 0.61±0.22, n=6 | t(10)=0.19; p=0.85; R^2^=0.004 |
| *Correlation with Damage Score* | |  |  |
| r=0.13, p=0.69 | r=-0.61, p=0.20 | *r=0.80, p=0.06* |  |
| Hippocampus | 1.08±0.21, n=6 | 0.76±0.15, n=5 | t(9)=1.19; p=0.27; R^2^=0.14 |
| *Correlation with Damage Score* | |  |  |
| r=-0.30, p=0.37 | r=0.01, p=0.98 | r=0.46, p=0.44 |  |
|  |  |  |  |
| *MAGL K_m_* | | | |
| Amygdala | 24.76±4.67, n=6 | 17.53±2.76, n=6 | t(10)=1.33; p=0.21; R^2^=0.15 |
| *Correlation with Damage Score* | |  |  |
| r=-0.23, p=0.47 | r=0.20, p=0.71 | r=0.07, p=0.89 |  |
| medial Prefrontal Cortex | 59.03±10.37, n=6 | 49.79±8.0, n=6 | t(10)=0.68; p=0.51; R^2^=0.04 |
| *Correlation with Damage Score* | |  |  |
| r=-0.12, p=0.70 | r=0.49, p=0.33 | r=-0.12, p=0.83 |  |
| Hypothalamus | 9.42±2.55, n=4 | 10.99±3.93, n=4 | t(6)=0.34; p=0.75; R^2^=0.02 |
| *Correlation with Damage Score* | |  |  |
| r=-0.23, p=0.58 | r=0.86, p=0.14 | r=-0.83, p=0.17 |  |
| Hippocampus | 24.82±4.51, n=6 | 23.03±4.57, n=6 | t(10)=0.28; p=0.79; R^2^=0.008 |
| *Correlation with Damage Score* | |  |  |
| r=-0.38, p=0.22 | r=-0.09, p=0.87 | r=-0.67, p=0.15 |  |

Effect of colitis on the binding affinity (K_m_) of anandamide (AEA) for its degradative enzyme fatty acid amide hydrolase (FAAH) and 2-arachidonylglycerol (AG) for its degradative enzyme monoacylglycerol lipase (MAGL).

Data are presented as mean ± standard error of the mean (SEM). *p<0.05, t-test saline versus trinitrobenzene sulfonic acid (TNBS).
