# Supplementary material for: Comorbid anxiety-like behavior in a rat model of colitis is mediated by an upregulation of corticolimbic fatty acid amide hydrolase
Source: Neuropsychopharmacology. 2021 Jan 15;46(5):992–1003. doi: 10.1038/s41386-020-00939-7 (PMC8115350; doi:10.1038/s41386-020-00939-7)
Supplement: Supplementary file 5 — Supplemental Table 4 [file 41386_2020_939_MOESM5_ESM.docx]

**Supplemental Table 4. Effects of TNBS-Induced Colitis on Endocannabinoid System Gene Expression.**

| **Amygdala** | | | |
| --- | --- | --- | --- |
| *Gene* | *Saline* | *TNBS* | *Statistics* |
| *Abhd4* | 1.00±0.26, n=5 | 0.61±0.05, n=7 | t(10)=1.76; p=0.11; R^2^=0.24 |
| vs. Damage | r=0.45, p=0.44 | r=0.59, p=0.18 | r=-0.38, p=0.23 |
| *Abhd6* | 1.00±0.21, n=7 | 0.77±0.05, n=7 | t(12)=1.10; p=0.29; R^2^=0.09 |
| vs. Damage | r=0.29, p=0.53 | *r=-0.74, p=0.06* | r=-0.34, p=0.23 |
| *Abhd12* | 1.00±0.14, n=7 | 0.93±0.12, n=7 | t(12)=0.37; p=0.72; R^2^=0.01 |
| vs. Damage | r=0.49, p=0.26 | r=0.05, p=0.92 | r=-0.08, p=0.79 |
| *Cnr1* | 1.00±0.1, n=7 | 1.01±0.11, n=7 | t(12)=0.1; p=0.92; R^2^=0.0008 |
| vs. Damage | r=0.33, p=0.47 | r=0.22, p=0.64 | r=0.09, p=0.75 |
| *Cnr2* | 1.00±0.23, n=7 | 0.88±0.14, n=7 | t(12)=0.43; p=0.67; R^2^=0.02 |
| vs. Damage | r=0.44, p=0.32 | r=0.43, p=0.33 | r=-0.02, p=0.95 |
| *Dagla* | 1.00±0.25, n=7 | 0.66±0.08, n=7 | t(12)=1.29; p=0.22; R^2^=0.12 |
| vs. Damage | r=0.44, p=0.33 | r=-0.35, p=0.44 | r=-0.16, p=0.59 |
| *Daglb* | 1.00±0.19, n=7 | 0.88±0.08, n=7 | t(12)=0.60; p=0.56; R^2^=0.03 |
| vs. Damage | r=0.44, p=0.33 | r=0.43, p=0.33 | r=-0.02, p=0.95 |
| *Faah* | 1.00±0.11, n=7 | 0.71±0.12, n=7 | *t(12)=1.79; p=0.10; R^2^=0.21* |
| vs. Damage | *r=0.76, p=0.05* | r=0.37, p=0.42 | r=-0.32, p=0.27 |
| *Fabp7* | 1.00±0.41, n=3 | 0.92±0.33, n=5 | t(6)=0.14; p=0.89; R^2^=0.003 |
| vs. Damage | r=0.55, p=0.63 | r=-0.03, p=0.96 | r=-0.06, p=0.89 |
| *Magl* | 1.00±0.28, n=5 | 0.67±0.12, n=6 | t(9)=1.18; p=0.27; R^2^=0.13 |
| vs. Damage | r=0.30, p=0.62 | r=-0.04, p=0.95 | r=-0.33, p=0.32 |
| *Napepld* | 1.00±0.14, n=7 | 1.12±0.11, n=7 | t(12)=0.69; p=0.50; R^2^=0.04 |
| vs. Damage | r=0.43, p=0.34 | r=0.05, p=0.92 | r=0.19, p=0.51 |
| *Trvp1* | 1.00±0.16, n=7 | 0.89±0.07, n=7 | t(12)=0.61; p=0.55; R^2^=0.03 |
| vs. Damage | r=0.57, p=0.18 | r=-0.04, p=0.93 | r=-0.16, p=0.59 |
|  | | | |

| **medial Prefrontal Cortex** | | | |
| --- | --- | --- | --- |
| *Gene* | *Saline* | *TNBS* | *Statistics* |
| *Abhd4* | 1.00±0.10, n=7 | 1.06±0.17, n=7 | t(12)=0.30; p=0.77; R^2^=0.007 |
| vs. Damage | r=-0.43, p=0.34 | r=-0.57, p=0.18 | r=-0.08, p=0.80 |
| *Abhd6* | 1.00±0.07, n=7 | 1.15±0.11, n=7 | t(12)=1.13; p=0.28; R^2^=0.10 |
| vs. Damage | r=0.15, p=0.75 | r=0.34, p=0.46 | r=0.38, p=0.18 |
| *Abhd12* | 1.00±0.12, n=7 | 0.99±0.04, n=7 | t(12)=0.08; p=0.94; R^2^=0.08 |
| vs. Damage | r=0.44, p=0.33 | r=-0.58, p=0.17 | r=-0.08, p=0.79 |
| *Cnr1* | 1.00±0.09, n=7 | 1.05±0.16, n=7 | t(12)=0.29; p=0.78; R^2^=0.007 |
| vs. Damage | r=0.50, p=0.25 | r=-0.05, p=0.92 | r=0.07, p=0.82 |
| *Cnr2* | 1.00±0.13, n=7 | 0.96±0.11, n=7 | t(12)=0.23; p=0.82; R^2^=0.004 |
| vs. Damage | r=0.10, p=0.83 | r=-0.43, p=0.33 | r=-0.15, p=0.61 |
| *Dagla* | 1.00±0.16, n=7 | 1.05±0.11, n=7 | t(12)=0.27; p=0.79; R^2^=0.006 |
| vs. Damage | r=0.58, p=0.17 | r=-0.07, p=0.88 | r=0.07, p=0.82 |
| *Daglb* | 1.00±0.21, n=7 | 0.82±0.02,n=6 | t(11)=0.75; p=0.47; R^2^=0.05 |
| vs. Damage | r=0.19, p=0.69 | r=0.09, p=0.87 | r=-0.21, p=0.49 |
| *Faah* | 1.00±0.13, n=7 | 0.96±0.05, n=7 | t(12)=0.30; p=0.77; R^2^=0.008 |
| vs. Damage | r=0.07, p=0.88 | *r=0.74, p=0.06* | r=0.004, p=0.99 |
| *Fabp7* | 1.00±0.26, n=7 | 0.63±0.06, n=7 | t(12)=1.38; p<0.19; R^2^=0.14 |
| vs. Damage | r=0.60, p=0.16 | r=-0.07, p=0.88 | r=-0.35, p=0.22 |
| *Magl* | 1.00±0.15, n=7 | 0.89±0.11, n=7 | t(12)=0.61; p=0.55; R^2^=0.03 |
| vs. Damage | r=0.65, p=0.11 | r=-0.04, p=0.93 | r=-0.17, p=0.57 |
| *Napepld* | 1.00±0.12, n=7 | 0.91±0.09, n=7 | t(12)=0.59; p=0.57; R^2^=0.03 |
| vs. Damage | r=0.39, p=0.39 | r=-0.45, p=0.31 | r=-0.24, p=0.41 |
| *Trvp1* | 1.00±0.1, n=7 | 0.84±0.10, n=7 | t(12)=1.09; p=0.30; R^2^=0.09 |
| vs. Damage | r=0.15, p=0.75 | r=-0.23, p=0.62 | r=-0.33, p=0.25 |
|  | | | |

| **Hypothalamus** | | | |
| --- | --- | --- | --- |
| *Gene* | *Saline* | *TNBS* | *Statistics* |
| *Abhd4* | 1.00±0.19, n=8 | 0.80±0.16, n=5 | t(11)=0.72; p=0.48; R^2^=0.05 |
| vs. Damage | r=-0.11, p=0.79 | *r=-0.88, p=0.05* | r=-0.37, p=0.21 |
| *Abhd6* | 1.00±0.17, n=8 | 0.77±0.14, n=5 | t(11)=0.97; p=0.35; R^2^=0.08 |
| vs. Damage | r=-0.01, p=0.98 | r=0.73, p=0.16 | r=-0.08, p=0.79 |
| *Abhd12* | 1.00±0.17, n=8 | 0.69±0.12, n=5 | t(11)=1.30; p=0.22; R^2^=0.13 |
| vs. Damage | r=-0.25, p=0.54 | *r=-0.88, p=0.05* | *r=-0.52, p=0.07* |
| *Cnr1* | 1.00±0.15, n=8 | 0.99±0.05, n=5 | t(11)=0.05; p=0.96; R^2^=0.0002 |
| vs. Damage | r=0.02, p=0.97 | r=0.33, p=0.59 | r=0.02, p=0.94 |
| *Cnr2* | 1.00±0.26, n=8 | 0.57±0.16, n=5 | ^ t(11)=1.2; p=0.26; R^2^=0.12 |
| vs. Damage | r=-0.11, p=0.79 | r=-0.26, p=0.68 | r=-0.34, p=0.26 |
| *Dagla* | 1.00±0.22, n=8 | 0.71±0.05, n=5 | t(11)=1.03; p=0.33; R^2^=0.09 |
| vs. Damage | r=-0.10, p=0.82 | *r=-0.93, p=0.02* | r=-0.32, p=0.29 |
| *Daglb* | 1.01±0.26, n=8 | 0.77±0.35, n=5 | t(11)=0.55; p=0.59; R^2^=0.03 |
| vs. Damage | r=0.35, p=0.39 | *r=0.84, p=0.07* | r=0.24, p=0.42 |
| *Faah* | 1.00±0.11, n=8 | 0.99±0.16, n=5 | t(11)=0.06; p=0.95; R^2^=0.0004 |
| vs. Damage | r=0.008, p=0.98 | r=-0.59, p=0.30 | r=-0.17, p=0.57 |
| *Fabp7* | 1.00±0.12, n=8 | 0.74±0.11, n=5 | t(11)=1.44; p=0.18; R^2^=0.16 |
| vs. Damage | r=0.10, p=0.82 | r=-0.38, p=0.53 | r=-0.32, p=0.29 |
| *Magl* | 1.00±0.16, n=7 | 0.95±0.28, n=5 | ^ t(10)=0.17; p=0.87; R^2^=0.003 |
| vs. Damage | r=0.31, p=0.50 | r=-0.41, p=0.50 | r=-0.07, p=0.83 |
| *Napepld* | 1.00±0.15, n=8 | 0.78±0.09, n=5 | t(11)=1.14; p=0.28; R^2^=0.11 |
| vs. Damage | r=-0.25, p=0.54 | r=-0.79, p=0.11 | r=-0.46, p=0.11 |
| *Trvp1* | 1.00±0.21, n=8 | 0.77±0.12, n=5 | t(11)=0.82; p=0.43; R^2^=0.06 |
| vs. Damage | r=-0.009, p=0.44 | r=-0.31, p=0.62 | r=-0.22, p=0.47 |
|  |  |  |  |

| **Hippocampus** | | | |
| --- | --- | --- | --- |
| *Gene* | *Saline* | *TNBS* | *Statistics* |
| *Abhd4* | 1.00±0.13, n=7 | 1.35±0.26, n=5 | ^ t(10)=1.35; p=0.21; R^2^=0.15 |
| vs. Damage | r=-0.36, p=0.43 | r=-0.08, p=0.90 | r=0.19, p=0.55 |
| *Abhd6* | 1.00±0.14, n=7 | 1.07±0.21, n=6 | t(11)=0.25; p=0.80; R^2^=0.25 |
| vs. Damage | r=-0.36, p=0.44 | r=0.11, p=0.84 | r=-0.02, p=0.94 |
| *Abhd12* | 1.00±0.12, n=7 | 1.12±0.11, n=6 | t(11)=0.75; p=0.47; R^2^=0.05 |
| vs. Damage | *r=-0.73, p=0.06* | r=0.13, p=0.80 | r=-0.08, p=0.80 |
| *Cnr1* | 1.00±0.13, n=7 | 1.43±0.47, n=6 | t(11)=0.93; p=0.37; R^2^=0.07 |
| vs. Damage | r=-0.14, p=0.76 | r=0.06, p=0.91 | r=0.21, p=0.49 |
| *Cnr2* | 1.00±0.23, n=7 | 1.24±0.32, n=6 | ^ t(11)=0.63; p=0.54; R^2^=0.03 |
| vs. Damage | r=-0.40, p=0.38 | r=-0.47, p=0.34 | r=-0.10, p=0.74 |
| *Dagla* | 1.00±0.14, n=7 | 0.92±0.17, n=6 | t(11)=0.38; p=0.71; R^2^=0.01 |
| vs. Damage | r=-0.42, p=0.35 | r=0.54, p=0.27 | r=-0.10, p=0.74 |
| *Daglb* | 1.00±0.07, n=6 | 1.05±0.15, n=6 | t(10)=0.27; p=0.80; R^2^=0.007 |
| vs. Damage | r=0.26, p=0.62 | r=0.22, p=0.68 | r=0.19, p=0.55 |
| *Faah* | 1.00±0.10, n=7 | 1.19±0.24, n=6 | t(11)=0.76; p=0.46; R^2^=0.05 |
| vs. Damage | r=0.10, p=0.83 | r=-0.05, p=0.92 | r=0.18, p=0.55 |
| *Fabp7* | 1.00±0.13, n=7 | 0.98±0.12, n=6 | t(11)=0.12; p=0.90; R^2^=0.001 |
| vs. Damage | r=-0.22, p=0.64 | r=0.05, p=0.93 | r=-0.10, p=0.73 |
| *Magl* | 1.00±0.15, n=6 | 1.24±0.15, n=6 | ^ t(10)=1.07; p=0.31; R^2^=0.10 |
| vs. Damage | r=0.28, p=0.59 | r=0.07, p=0.90 | r=0.32, p=0.31 |
| *Napepld* | 1.00±0.06, n=6 | 1.82±0.51, n=6 | t(10)=1.60; p=0.14; R^2^=0.20 |
| vs. Damage | *r=-0.74, p=0.09* | r=0.23, p=0.66 | r=0.39, p=0.22 |
| *Trvp1* | 1.00±0.25, n=7 | ^ 0.79±0.17, n=5 | t(10)=0.65; p=0.53; R^2^=0.04 |
| vs. Damage | r=0.13, p=0.77 | r=-0.548, p=0.41 | r=-0.17, p=0.60 |

Effects of colitis on expression of genes related to the endocannabinoid system.

Data are presented as mean ± standard error of the mean (SEM). Two-tailed t-test saline versus trinitrobenzene sulfonic acid (TNBS). Pearson correlations with macroscopic tissue damage.

^ Indicates threshold (C_t_) values greater than 30, but less than 40, which indicated less than abundant to weak expression.

α,β-hydrolase 4 (ABHD4), α,β-hydrolase 6 (ABHD6), α,β-hydrolase 12 (ABHD12), Cannabinoid receptor 1 (CB1), Cannabinoid receptor 2 (CB2), Diacylglycerol lipase (DAGL)α, Diacylglycerol lipase (DAGL)β, Fatty acid amide hydrolase (FAAH), Fatty acid binding protein (FABP)7, Monoacylglycerol lipase (MAGL), *N*-acyl phosphatidylethanolamine-specific phospholipase D (NAPE-PLD) and transient receptor potential cation channel subfamily V member 1 (TrpV1).
