# Supplementary material for: Comorbid anxiety-like behavior in a rat model of colitis is mediated by an upregulation of corticolimbic fatty acid amide hydrolase
Source: Neuropsychopharmacology. 2021 Jan 15;46(5):992–1003. doi: 10.1038/s41386-020-00939-7 (PMC8115350; doi:10.1038/s41386-020-00939-7)
Supplement: Supplementary file 6 — Supplemental Figure 1 [file 41386_2020_939_MOESM6_ESM.pdf]

Figure S1

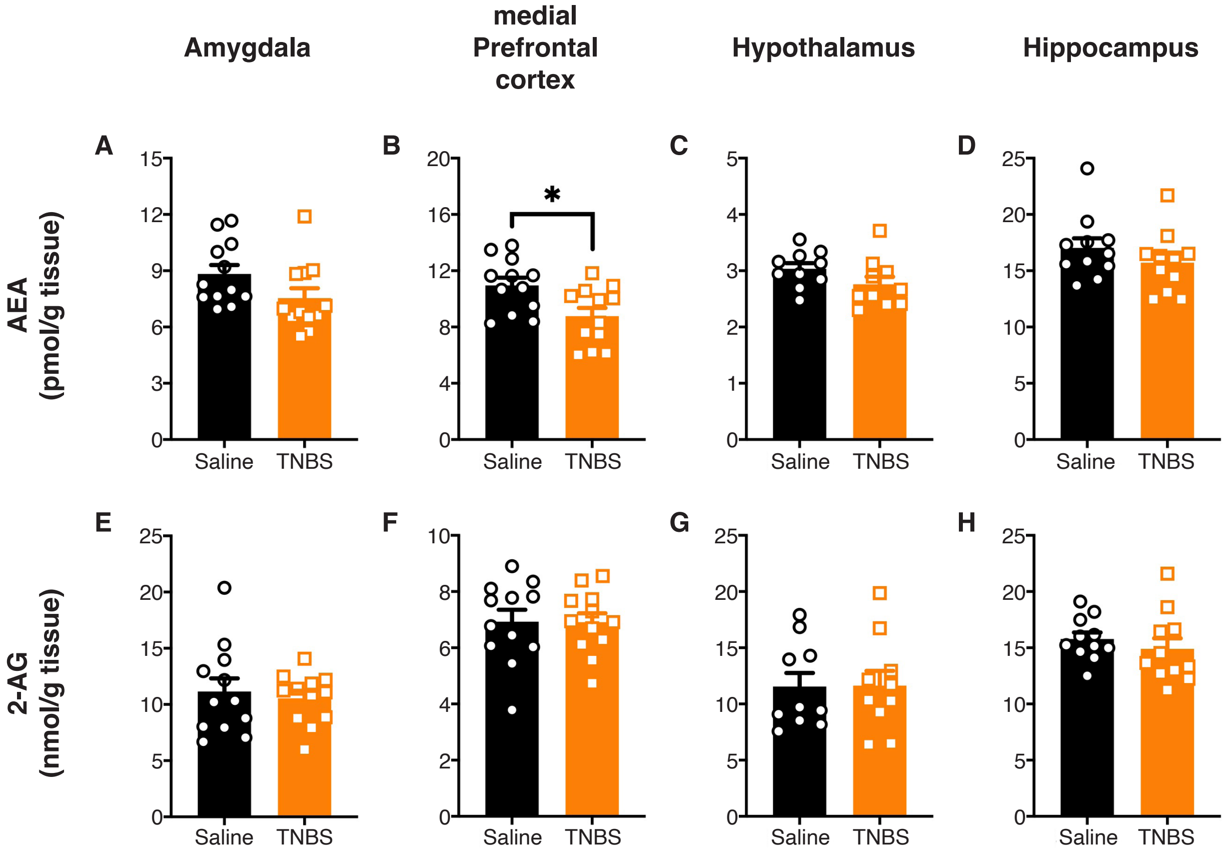

Figure S1. Colitis alters AEA but not 2-AG levels in females.

In female rats exposed to colitis, there was a reduction in AEA levels in the (B) medial prefrontal cortex and potentially in the (A) amygdala, but not in the (C) hypothalamus and (D) hippocampus. 2-AG levels in the (E) amygdala, (F) medial prefrontal cortex, (G) hypothalamus or (H) hippocampus were not altered following exposure to TNBS induced colitis.

n=10-12/group. * $p<0.05$, two-tailed t-test saline versus TNBS.

Saline=left, black bars with circles. TNBS=right, orange bars with squares.
